# Thoracic dysfunction in whiplash associated disorders: A systematic review

**DOI:** 10.1371/journal.pone.0194235

**Published:** 2018-03-23

**Authors:** Nicola R. Heneghan, Richard Smith, Isaak Tyros, Deborah Falla, Alison Rushton

**Affiliations:** 1 Centre of Precision Rehabilitation for Spinal Pain, School of Sport, Exercise & Rehabilitation Sciences, University of Birmingham, Birmingham, United Kingdom; 2 Department of Allied Health Professions, University of the West of England, Bristol, United Kingdom; 3 Edgbaston Physiotherapy Clinic, Birmingham, United Kingdom; Taipei Veterans General Hospital, TAIWAN

## Abstract

**Background:**

Research investigating Whiplash Associated Disorder (WAD) has largely focused on the cervical spine yet symptoms can be widespread. Thoracic spine pain prevalence is reported ~66%; perhaps unsurprising given the forceful stretch/eccentric loading of posterior structures of the spine, and the thoracic spine’s contribution to neck mobility/function. Approximately 50% WAD patients develop chronic pain and disability resulting in high levels of societal and healthcare costs. It is time to look beyond the cervical spine to fully understand anatomical dysfunction in WAD and provide new directions for clinical practice and research.

**Purpose:**

To evaluate the scope and nature of dysfunction in the thoracic region in patients with WAD.

**Methods:**

A systematic review and data synthesis was conducted according to a pre-defined, registered (PROSPERO, CRD42015026983) and published protocol. All forms of observational study were included. A sensitive topic-based search strategy was designed from inception to 1/06/16. Databases, grey literature and registers were searched using a study population terms and key words derived from scoping search. Two reviewers independently searched information sources, assessed studies for inclusion, extracted data and assessed risk of bias. A third reviewer checked for consistency and clarity. Extracted data included summary data: sample size and characteristics, outcomes, and timescales to reflect disorder state. Risk of bias was assessed using the Newcastle-Ottawa Scale. Data were tabulated to allow enabling a semi-qualitative comparison and grouped by outcome across studies. Strength of the overall body of evidence was assessed using a modified GRADE.

**Results:**

Thirty eight studies (n>50,000) which were conducted across a range of countries were included. Few authors responded to requests for further data (5 of 9 contacted). Results were reported in the context of overall quality and were presented for measures of pain or dysfunction and presented, where possible, according to WAD severity and time point post injury. Key findings include: 1) high prevalence of thoracic pain (>60%); higher for those with more severe presentations and in the acute stage, 2) low prevalence of chest pain (<22%), 3) evidence of thoracic outlet syndrome, with some association to and involvement of the brachial plexus, 4) muscle dysfunction in the form of heightened activity of the sternocleidomastoid or delayed onset of action of the serratus anterior, 5) high prevalence of myofascial pain and trigger points in the scalene muscles, sternocleidomastoid and mid/lower fibres of trapezius muscle (48–65%), and 6) inconclusive evidence of altered thoracic posture or mobility.

**Conclusions:**

Considerable evidence supports thoracic pain and dysfunction in patients with WAD, involving primarily nerves and muscles. Notwithstanding the low/very low level of evidence from this review, our findings do support a more extensive clinical evaluation of patients presenting with WAD. Additional high quality research is required to further characterise dysfunction across other structures in the thoracic region, including but not limited to the thoracic spine (mobility and posture) and thoracic muscles (stiffness, activation patterns). In turn this may inform the design of clinical trials targeting such dysfunction.

## Introduction

The cumulative incidence of patients seeking healthcare post-whiplash from a road traffic accident has increased over the last 30 years to an annual incidence of between 3 and 6/1000 inhabitants in North America and Western Europe [1]. Following injury, individuals experience a range of clinical manifestations, described as Whiplash Associated Disorder (WAD), including neck pain, fatigue, nausea, low self-reported physical and mental health, cognitive impairments and pain in multiple sites [2]. The severity of presentation in WAD is categorised according to the Quebec Task Force Classification (QTF) where the presence of clinical signs and symptoms relate to the severity of the disorder [3].

Whilst research has identified risk factors for poor prognosis [4, 5], and enhanced understanding of neurophysiological changes [6], it is not known why disability and pain persist beyond normal tissue healing times. With 40–60% patients progressing to experience chronic whiplash associated disorder (CWAD), estimated costs of ~$4 billion (USA) and ~€10 billion (Europe) associated with management and time off work [7, 8], further research is needed to fully understand anatomical dysfunction in WAD and provide new directions for clinical practice and research. This includes the effects on anatomically related body regions, such as the thoracic spine. Potential ongoing dysfunction in the thoracic region may partially explain why there is inconclusive evidence for the effectiveness of physiotherapy management for WAD II, where interventions target a primary complaint of neck pain [9, 10].

Although current research into WAD has focused on the primary complaint of neck pain [11], symptoms may also include stiffness [12, 13] and pain in other regions including the jaw, head, upper and lower limbs, chest, abdomen and groin [14]. Moreover, data from a large cohort study (n = 6481) reported that 66% of individuals complained of thoracic mid-spine pain post whiplash injury, with 23% still experiencing symptoms one year later [14]. This is not surprising given the mechanism of a whiplash injury which involves forceful stretch loading to the upper back muscles; muscles which span both the neck and thoracic spine [15]. Recent evidence supports the presence of pathology in the mid/lower fibres of the trapezius muscle where they insert onto bone (myofascial-entheseal dysfunction) [16], which may partly account for the high prevalence of thoracic pain reported in both acute (65.5%) [14] and >80% in chronic WAD [17]. Furthermore, a number of abnormalities have been documented for the trapezius muscle in people with chronic WAD including histological changes as well as changes in muscle behaviour [18, 19].

With reduced cervical mobility being characteristic of chronic WAD and evidence that the thoracic spine contributes up to 33% and 21% of the movement occurring during neck flexion and rotation respectively [20] perhaps thoracic mobility is impaired following a whiplash injury; however at this time relatively little is known about the impact of WAD on this spinal region [21]. Research is now needed to examine the impact of injury on the thoracic spine in WAD.

Nevertheless, a systematic review of the current evidence has never been conducted to examine the scope and nature of dysfunction/impairment in the thoracic spine region following whiplash injury and in WAD. Knowledge of such dysfunction may be used to inform clinical practice and examination of patients with WAD, but also future clinical trials of novel interventions targeting thoracic impairments in WAD.

### Objectives

The primary objective is to evaluate the scope and nature of dysfunction in the thoracic spine region in patients with WAD. A secondary objective is to explore the scope and nature of such changes based on severity using the Quebec Task Force classification (I-III) and stage post injury (acute/sub-acute less than 3 months or chronic 3+ months). Thirdly, we wish to make evidence based recommendations for clinical practice and future research.

## Methods/Design

### Protocol and registration

A systematic review of all forms of observational study was conducted according to a pre-defined protocol [22], in line with the Centre of Research and Dissemination Guidelines [23], Meta-analyses of Observational Studies in Epidemiology (MOOSE) [24] and is reported in line with Preferred Reporting Items for Systematic Reviews and Meta-Analyses (*PRISMA*) [25], S1 Table. PROSPERO (Registration number: CRD42015026983).

### Eligibility criteria

Eligibility criteria informed using SPIDER [26], included that the sample (S) comprised patients aged >19 years; the phenomenon of interest (PI) was a WAD following motor vehicle or sporting injury; investigated using an observational study design (cohort, case control, single case study) (D) with evaluation of patient reported or performance based measure(s) of thoracic dysfunction of one or more of the following: muscle with an insertion to the thoracic cage, bone or joint of the thoracic cage, neural tissue related to the thorax (E).

Exclusion criteria included: studies investigating upper trapezius, studies investigating a central pain mechanism or neurophysiology of pain where no testing took place in the thoracic region, simulation or modelling studies, fractures (WAD IV), visceral injury or fibromyalgia.

### Information sources

The search employed sensitive topic-based strategies designed for each database from inception to 1/6/16. No language or geographical restrictions were included. Databases included, CINAHL, EMBASE, MEDLINE, ZETOC, Index to Chiropractic Literature ChiroAccess and Google Scholar. Selected Internet sites and Indexes including, Turning Research into Practice, PubMed, National Research Register and Cochrane Back Review Group were also searched. Hand searching of key journals included Spine and the European Spine Journal. Grey literature included British National Bibliography for Report Literature, Dissertation Abstracts, Index to Scientific and Technical Proceedings, National Technical Information Service and the System for Information on Grey Literature.

### Search strategy

The search strategy included terms related to whiplash associated disorder and patient reported or performance based measures of thoracic dysfunction. Terms and keywords derived from the scoping search and experts [subject specific (NRH,AR) and methodological (NRH,AR)] included: ‘whiplash’, ‘whiplash associated disorder’, ‘WAD’, ‘whiplash injury’, ‘motor vehicle accident OR collision’, ‘road traffic accident’, ‘cervical strain’ and ‘thoracic spine’, ‘dorsal spine’, ‘mid-spine’, ‘thoracic injuries’, limiting to adults >19 years and diagnosis to achieve the best balance of sensitivity and specificity. An example a search from Medline is included S2 Table. Terms were adapted to reflect spelling differences and [14] unique searching features of individual databases. Reference lists of included papers were also searched.

### Study selection

Two reviewers (NRH, RS) independently searched information sources and assessed identified studies for inclusion, facilitated by grading each eligibility criterion as eligible/not eligible/might be eligible [27]. Full texts were reviewed and included when both reviewers agreed [23]. A third reviewer (IT) mediated in the event of disagreement [28].

### Data collection process and items

Using a standardised form, the two reviewers extracted data independently [23]. A further reviewer (IT) independently examined data for accuracy and clarity. Authors were contacted for additional information or data where required.

### Data items

Data were extracted from each study, including: study design, sample characteristics including age, gender, severity of WAD using the QTF Classification if reported, time point post injury and patient reported or performance based measures of thoracic dysfunction.

### Risk of bias in individual studies

Risk of bias for each included study was independently assessed by the same initial reviewers. The third reviewer mediated in situations of disagreement. All tools and processes were piloted prior to use. Risk of bias was only assessed for cohort and case-control studies using the Newcastle-Ottawa Scale [29]; that includes eight items that are rated and categorised into three groups, namely selection, comparability and outcome.

### Summary measures

Summary measures of patient reported or performance based measures of thoracic dysfunction are presented in the form of prevalence data and confidence intervals where provided, for thoracic pain, chest pain, thoracic outlet syndrome (TOS), myofascial pain and trigger points, dysfunction involving the brachial plexus, thoracic spine posture or mobility. Results are presented where possible, according to severity (QTF) and stage of WAD i.e. acute/sub-acute (< 3 months) or chronic (> 3 months).

### Synthesis of results

In accordance with the protocol [22] meta-analyses would be performed where a sufficient number of studies share all of the stated characteristics; design, measure of dysfunction, severity based on QTF and stage post whiplash injury.

### Quality of evidence across studies

Quality of evidence, including risk of bias across studies was evaluated using GRADE [30] for individual outcomes of interest. By their very nature, observational studies are considered ‘low quality’ although could be *upgraded* where a large dose response was evident, or the effects could not be accounted for by bias [30]. Likewise, findings could be downgraded to ‘*very low*’ where concerns were identified from the body of studies relating to precision, consistency, directness, precision or potentially other additional domains relating to strength of association e.g. magnitude of effect [30].

## Results

### Study selection

**Fig 1:**
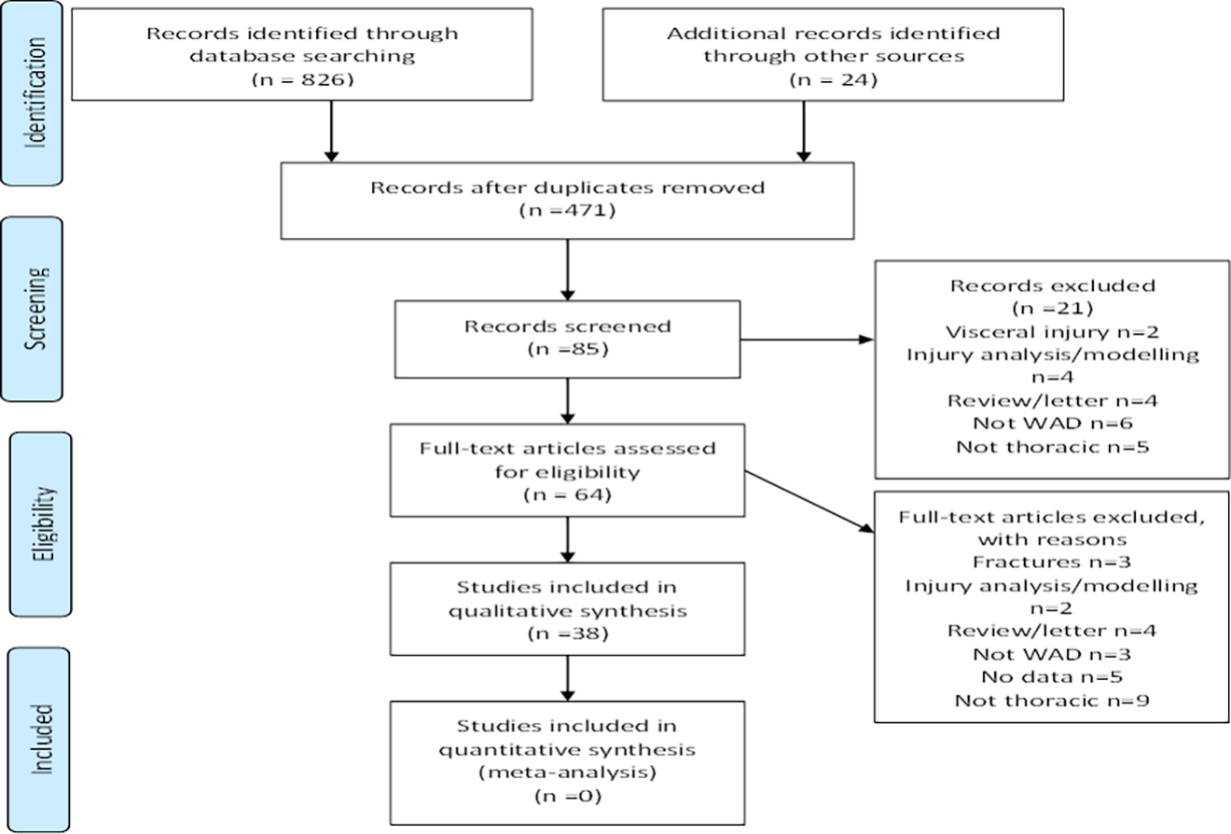
Flow chart of search and study selection.

A total of 38 studies met the eligibility criteria, including 19 cohort studies, 16 case control studies and 3 single case studies/reports. The process of selection is detailed in Fig 1, with the list of excluded studies and reasons provided S3 Table.

### Study characteristics

Study characteristics are detailed in Table 1. A range of measures of thoracic dysfunction were identified, including thoracic pain, chest pain, involvement of the brachial plexus, thoracic outlet syndrome, changes in muscle activation *etc*. An overview of the types of dysfunction is provided in Table 2 and, where possible, is presented according to severity and stage following injury.

**Table 1:** Study characteristics.

Author & Date	Design	WAD patients	Age Gender	Sample	Assessment	Findings
Alexandre 2005[31]	Cohort	‘**Mild car accidents**’, mainly rear shunts **Chronic:** 2–48 months post injury	Mean (range) age 36.5 (19–57) years • Female n = 20 • Male n = 4	N = 24	• Clinical examination • X-ray of the cervical spine and ‘possibly MRI or CT scan’ • Electrodiagnostic testing	**Thoracic outlet syndrome due to brachial plexus entrapment** • Right side n = 13 • Left side n = 9 • Bilaterally n = 9
Berglund2001[32]	Case control although stated cohort design applied?	**Minor WAD** (including n = 1 severe, n = 4 moderate) **Chronic:** 7 years +	No data	N = 4124 • Exposed with WAD = 242 • Exposed without WAD = 204 • Unexposed comparison = 3688	Postal questionnaire	**Thoracic pain prevalence post rear end collision** (%, 95% CI, and number) • **Injury with WAD: 15.3% (10.4–21.5), 27/176** • Injury without WAD: 6.7% (3.1–12.3), 9/135 • No injury: 4.4% (3.3–5.8), 52/1173
Bismil 2012[16]	Cohort	**Chronic WAD II** (>6 mths)	For sub group: mean age 42 years • Female 56% • Male 44%	N = 1025 with sub group n = 25	Clinical examination	**Myofascial-entheseal dysfunction** • Bilateral 60%; unilateral 40% • Middle trapezius 56% • Lower trapezius 12% **Enthesopathy** (myofascial pain and trigger points) n = 25 • middle and lower parts of trapezius • scapular attachment • 48%
Bock2005[33]	Cohort (reliability study)	**Chronic WAD** (>3 mths) • 11/31 WADI • 20/31 WADII	Mean, range (SD) age 40.9, 16–72 (14.8) years • Females n = 17 • Males n = 5	N = 31, (9 excluded)	Clinical examination	**Thoracic allodynia** 71% of sample • Lower thoracic segments are most commonly involved
Bodack 1998[34]	Case report	**Acute WAD(?III)**	19 years Female	N = 1	• Clinical examination • Electrodiagnostic studies	**Upper back pain** • Weak mid and lower trapezius
Bortsov2014[11]	Cohort 8 Emergency Depts	**Mild** (99% AIS1) **WADI & II** (**acute, sub-acute, chronic**)	Age tertiles at baseline • 18–26 years n = 315 • 27–41 years n = 319 • 42–65 years n = 314	N = 948 completed baseline evaluations • -859/948 (91%) fu at 6 weeks, • -839/948 (89%) fu 6 months • -861/948 (91%) fu at one year	Evaluation via telephone interview or a web-based questionnaire.	**Thoracic and chest pain** • **47% prevalence of upper back pain** at 6 weeks (18% being widespread. 29% localised) • **19% prevalence of chest pain** at 6 weeks (9% being widespread, 10% localised) *No data available for other time points*, *authors contacted*
Capistrant1976[35]	Cohort	**WAD with TOS** • **Acute** n = 14**Chronic** n = 16	Mean (range) age 40 (21–59) years • Female n = 17 • Male n = 13	N = 35 TOS group n = 30	• Cervical x-ray • Clinical examination • Electrodiagnostic testing	**Thoracic outlet syndrome** • Unilateral symptoms n = 25 • Bilateral symptoms n = 5
Capistrant1986[36]	Cohort	**WAD** (including cervical strains)	Mean age 30 years • Females n = 32 Males n = 8	N = 111	• Clinical examination • Electrodiagnostic studies	**Thoracic outlet syndrome** • TOS n = 40/111, 36%
Castaldo2014[37]	Case control	**Chronic WADII & III** Control (Mechanical neck pain)	Mean (SD) age41.6 (1.72) • Females n = 28 • Males n = 21	N = 49	Clinical examination	**SCM Myofascial trigger points** • Latent MTP left 13, right 15 • Active MTP left 21, right 22 **MNP group:** • Latent MTP left 6, right 9 • Active MTP left 13, right 13
Chien 2009 [38]	Case control	**Chronic WADII** (3mths-3 years)	**WAD** Mean (SD) age 35.3 (10.7) years Females n = 25	WAD N = 31	Clinical examination	**BPTT (from 180-degree extension elbow)** • WAD -22.3 (27.4) degrees VAS 2.4 (2.3) • Control -11 (5.9) VAS 0.7 (1.1)
			**Control** Mean (SD) age 31.4 (8.9) years Females n = 25	Control N = 31		
Cornips2014 [39]	Case series of surgical cases for TDH	**Chronic WAD**	Range age 26–58 • Females n = 7 • Males n = 3	N = 10 (N = 4 had ‘typical whiplash based on MOI & complaints)	Clinical examination and imaging (from 326 discectomies for ≥ 1 thoracic disc herniation)	**Thoracic pain (local) with thoracic disc herniation** N = 10, ranging from significant axial pain to paretic-paralytic (significant motor weakness) • Significant axial and thoracic radicular pain n = 1 • Significant axial and lower leg pain with or without thoracic radicular pain n = 3
Ettlin2008[40]	Case control	**Chronic WAD** (with or without neurological deficit were included)	**WAD** Mean (SD) age 38.57 (10.18) years Females n = 35 (74.5%)	WAD N = 47	Clinical examination	**Myofascial trigger points scaleneus medius and SCM)** Prevalence (%) **WAD** • Scaleneus medius 30 (63.8) • SCM 24 (51.1) **Control** • Scaleneus medius 3 (12.5) • SCM 3 (12.5)
			**Control** Mean (SD) age 37.42 (11.34) years Females n = 1 (45.8%)	Control N = 24		
Fernandez-Perez2012 [41]	Case control	**Sub-acute WADII** *(states acute in text)*Within 1 month	**WAD** Mean (SD) age 28.7 (12.4) years	WAD N = 20	Clinical examination	**Myofascial trigger points (scaleneus medius and SCM)** Number of participants with MTP **WAD** • Scalene Active R 6, L4, Latent R 9, L10, No R 5, L6 • SCM Active R1, L6, Latent R12, L9, No R7, L5 **Control** • Scalene Active R0, L0, Latent R2, L4, No R 18, L16 • SCM Active R0, L0, Latent R4, L5, No R 6, L15
			**Control** Mean (SD) age 29.1 (12.2) years	Control N = 20		
Ferrari2010 [42]	Cohort	**WADI & II (acute, sub-acute)**Presented at 7 days, FU at 3 months	Mean, range (SD) age 37.5, 18–71 (13) years • Female n = 37 • Male n = 32	N = 69	Clinical examination	**Brachial plexus provocation test (Pain and elbow ROM)** **3 months:** • VAS: mean (SD)2.2 (1.2) • Elbow extension: 41.5 (23) degrees **Expectation predicted elbow angle and VAS on BPPT at 3 months** Significantly smaller angle when expected to ‘get better slowly’ or ‘get better soon’ vs the other 2 categories VAS for ‘get better soon’ 1 point less than other 3 groups
Hartling2002[43]	Cohort	**WADI-III**<2 weeks and 6 mths	No details	N = 380	Clinical examination	**Upper back pain** <2 weeks: 74.6% adjusted OR 2.91 (1.65,5.12) ***Symptoms intensity OR (95%CI)*** • *Mild 3*.*09 (1*.*50*, *6*.*38)* • *Moderate 4*.*17 (2*.*08*, *8*.*36)* • *Severe 15*.*63 (6*.*07*, *41*.*51)* ***Symptoms frequency OR (95%CI)*** • *Mild 3*.*47 (1*.*25*, *9*.*66)* • *Moderate 5*.*36 (2*.*84*, *10*.*17)* • *Severe 5*.*30 (2*.*53*, *11*.*18)*
Helgadottir2011a[44](sub group of Helgadottir, 2011)	Case control	**Chronic WADII** (>6 mths)	**WADII** Mean (SD) age 33.37 (9.58) years NDI 38 (18.74) • Female n = 20 Male n = 3	WAD N = 23	Clinical examination	In WAD group: No difference was found in the **mid thoracic curve** (*p* = .99)
			**Control** Mean (SD) 29.70 (7.75) years • Female n = 17 • Male n = 3	Control N = 20		
Helgadottir2011b [45]	Case control	**Chronic WADII** (>6 mths)	**WADII** Mean (SD) age 33 (10) years VAS 6(2) NDI 38 (18) • Female n = 24 Male n = 3	WAD N = 27	Electromyography	**Muscle activation and duration** (middle trapezius, lower trapezius and serratus anterior) • **Delayed onset of serratus anterior muscle** activation in the WAD group (P < .01) • **Reduced duration of muscle activity** in the WAD group (P < .01) • No change in in middle and lower trapezius
			**Control** Mean (SD) 30 (8) years • Female n = 18 • Male n = 5	Control N = 23		
Hincapie2010 [14]	Cohort	**WAD**<30 days post traffic injury	Mean (SD) age37.2 (15.2) years • Female 60.2% • Male 39.8%	N = 6481	Clinical examination	**Mid back pain** • Prevalence % (95%CI) 65.5 (64.4–66.7) • Localised % (95%CI) 0.06 (0.00–0.12) **Posterior shoulder (includes upper thoracic)** • Prevalence % (95%CI) 75.3 (74.3–76.4) **Chest pain** • Prevalence, % (95%CI) 18.9 (17.9–19.8) • Localised % (95%CI) 0.05 (0.00–0.10)
Holm2007[17]	Cohort	**WAD** Approx. 10 days to 6 months	Age n (%)≥40 n = 21 (21) • 30–39 n = 18 (22) • 18–29 n = 17 (20) • Females = 38 (24%)	N = 56	Clinical examination	**Chest pain prevalence, n (%)** • Baseline - • 6-weeks 2(6) • 4-months 1(4) • 8-months 2(12) • 12-months 5(22) **Thoracic pain prevalence, n (%)** • Baseline 29 (52) • 6-weeks 28 (80) • 4-months 24 (86) • 8-months 16 (94) • 12-months 20 (87)
Ide2001 [46]	Cohort	**WAD** 1week to 6 months	Mean, range age • Males = 36.1, • Females = 35.7 • 14–61 years	N = 119	• Cervical radiographs • Clinical examination	**Non-brachial plexus irritation n = 74** **Brachial plexus irritation n = 45**
Johansson2015[2]	Cohort from a large population based study	**WAD**	Median age35.7 (25.3–47.2) years Females = 2484 (66.9%)	N = 3711 from 8634 eligible cases	Clinical examination	**Mid back pain present in 3711 individuals (43%)** • Incidence 236/100,000 • Baseline pain rating 6 (5–8) • 23% not recovered at one year
Kai2001[47]	Cohort	**Cervical strain** post cervical trauma	No data reported	N = 110	• Clinical examination • MRI	Cervical strain n = 29 (no positive tests) • ?Neurogenic TOS n = 25 (one positive test) • Neurogenic TOS n = 39 (2 or 3 positive tests) • Neurogenic TOS with associated cervical disc disease (n = 17) **Pain (scapular)** • Cervical strain 31% • Probable Neurogenic TOS 30% • Neurogenic TOS 46% • Neurogenic TOS with associated cervical disc disease 35%
Kall 2008[48]	Longitudinal study using data from previous RCT (use baseline data) Cohort study applied	**WADI-II (sub-acute)** (96% MVAs, 4% falls)	Mean (range)age 31 (18–61) years • Female n = 30 (64%) • Male n = 17 (36%)	N = 47	Clinical examination	Women with sub-acute WAD C7-T1 flexion hypomobility *No baseline data available*, *authors contacted*
Klein2001 [49]	Case control	**Chronic WADI-III** (mainly II)>3 months	**WAD** Mean (SD) 36.4 (10.1) years • Female 67% • Male 33% **Control**	WAD N = 46	• Clinical examination • Electromyography	**SCM activation** • No earlier activation during cervical rotation in CWAD (Cervical ROM WAD 84.8 (31)-degrees compared with 137.2 (18.5)-degrees in control)
			Mean (SD) 28.8 (6.7) years • Female 58% • Male 42%	Control N = 48		
Koelbeck 1999 [50]	Case control	**Chronic WADII**	**WADII** Mean (range) 42 (28–69) years Female n = 7 Male n = 4 **Control** Mean (range) 39 (26–50) years Female n = 7 Male n = 4	WAD N = 11 Control N = 11	Clinical examination	**Infraspinatus region (WAD n = 8)** **Pressure pain threshold (kPa)** Mean (SD) • WAD 152.2 (84.9) • Control 492.8 (18.8) **Pin prick thresholds** Mean (SD) • WAD 11.5 (2.5) • Control 12.4 (1.1) **VAS score (cm)** Mean (SD) • WAD 5.2 (0.9) • Control 5.3 (0.4) **VAS area (cm sq)** • WAD 4138.1 (1707.2) • Control 780.5 (366.3) **VAS peak (cm)** • WAD 8.7 (1.5) • Control 5.2 (1.8) **VAS onset (s)** • WAD 22.7 (6.5) • Control 35.5 (5) **VAS duration (s)** • WAD 719.5 (244.8) • Control 317.7 (115.4)
Magnusson 1994 [51]	Cohort	**Chronic WADI-III** (>6 mths)	• Mean (range) age33 (17–52) yearsFemales n = 25 (65.8%) • Males n = 13 (34.2%)	N = 38	• Cervical radiographs • Clinical examination	**Thoracic outlet syndrome** n = 12 (31.6%) • Unilateral n = 9 (23.7%) • Bilateral n = 3 (7.9%) (N = 4 had symptoms, but not reproducible on palpation)
McLean, 2014 [52]	Cohort	**WADI & II** Data for **acute** (<24 hours injury) and **sub-acute**(6 weeks)	Median (range) age36 (18–65) years Females 60.7% (Females 62.6% 6 weeks?sub-acute)	Acute N = 948 Chronic n = 711 (non-litigant group) 6 weeks: N = 859	Participant interview	**Pain (moderate or severe NRS 4)6 weeks** • Upper back 21% (18–24%) • Shoulder (including posterior upper thoracic) 23% (20–26%) • Chest 8% (6–10%) *No data available for other time points*, *authors contacted*
Myran2011[53]	Cohort	**Chronic WAD**	Age Mean across subgroups 40.8–56.0) Female 44.3–67.1%	N = 46,895	Questionnaire	**Chest/abdomen** **Men** Total 972 • N = (%) 68 (7.0) • OR (95%CI) 3.6 (2.4, 5.2) **Women** Total 1543 • N = (%) 107 (6.9) • OR (95%CI) 7.1 (4.9, 10.4) **Upper back** **Men** Total 1421 • N = (%) 125 (8.8) • OR (95%CI) 5.0 (3.7, 6.7) **Women** Total 3361 • N = (%) 235 (7.0) • OR (95%CI) 5.9 (4.4, 7.8)
Omar, 2007[54]	Case report	**Chronic WADIII**(6 months)	30 year old male	N = 1	• Clinical examination. • MRI • Electromyography • Nerve conduction studies	**Left brachial plexus lesion** • Denervation of L serratus anterior and reduced motor unit recruitment (Winging scapular)
Sterling 2002 [55]	Case control	**Chronic WADII & III**>3months	**WAD** • Mean (SD) 37.43 (9.3) yearsFemale n = 127 • Male n = 29	WAD N = 156	Clinical examination	**Brachial plexus provocation test** **ROM** (from -180degrees) Mean (95%CI) • WAD -26.21 (-28.05, -24.37) • Control -12.92 (-15.24, -10.6) **VAS (0–10)** • WAD 4.93 (4.6, 5.3) • Control 2.62 (2.2, 3.04) **WAD vs control** Significantly higher VAS and less ROM on elbow extension in WAD group (p<0.001) **Subgroups within WAD:** • Group 1: Whole arm pain reproduced with BPPT n = 40 • Group 2: Arm pain not reproduced with BPPT n = 54 • Group 3: No arm pain n = 62 Elbow extension significantly less in group 1 then group 2 Elbow extension significantly less and VAS significantly higher in group 1 vs group 3 No difference in ROM or VAS between group 2 and 3
			**Control** Mean (SD) 38.95 (14.47) years • Female n = 50 • Male n = 45	Control N = 95		
Sterling 2003a [56]	Case control	**WADII-III** (< 1 month)	**WAD** Mean (SD) 36.27 (12.69) years • Female n = 45 • Male n = 21	WAD N = 66	• Clinical examination • Electromyography	**SCM activity** Increased SCM muscle activity across all point and disability ratings with higher disability resulting in heighten SCM activity • **Recovered group** n = 25, NDI<8, 29±4% • **Mild pain and disability** n = 22 NDI 10–28, 27±3% • **Moderate/severe pain and disability** n = 19, NDI >30, 40±4% • **Control** n = 20, 16±3%
			**Control** Mean (SD) 40.1 (13.6) years • Female n = 12 • Male n = 8	Control N = 20		
Sterling 2003b(?same as 2003a)[57]	Case control	**Sub-acute WADII-III**	**WAD** Mean (SD) 36.27 (12.69) years • Female n = 56 • Male n = 24	WAD N = 80	Clinical examination	**BPTT (from 180-degree extension elbow)** • **<1 months post injury** • **Recovered group** -23.95 (2.4) degrees, VAS 1.8 (04) • **Mild pain** -33.97 (2.6) degrees, VAS 3.2 (0.5) • **Moderate/severe pain** -34.27 (3.4) degrees, VAS 4.1 (0.5) • **Control** -20.67 (3.12) degrees, VAS 1.8 (04) **Mild pain** recovered and no different from controls at 2 months **6 months** **Moderate/severe pain:** continued to have higher VAS 3.4 (1.0) and reduced elbow extension (no data provided)
			**Control** Mean (SD) 40.1 (13.6) years • Female n = 12 • Male n = 8	Control N = 20		
Sterling 2004 [12]	Case control	**Acute WADII-III**<1 month	**WAD** Mean (SD) 33.5 (14.7) years • Female n = 56 • Male n = 24	WAD N = 80	• Clinical examination • Electromyography	**SCM activity (%)** • **Control** 13(3)% • **Mild** 32(3)% • **Moderate** 29(4)% • **Severe** 47(4)% **BPTT [mean (SD) from 180-degree extension elbow + VAS mean (SD)]** • **Control** -21.4 (10.8), VAS 1.7 (1.7) • **Mild** -26.7 (17.7), VAS 1.8 (1.7) • **Moderate** -31.3 (14.9), VAS 3.0 (1.8) • **Severe** -36.5 (11.8), VAS 4.3 (1.6) Significantly decreased elbow extension ROM and higher VAS in severe pain group versus the other 2 whiplash groups
			**Control** Mean (SD) 39.5 (14.6) years • Female n = 11 • Male n = 9	Control N = 20		
Sterling 2005 (same population 2004)[58]	Cohort	**Chronic WADII-III** (96% WAD II)	**WAD** Mean (SD) 36.27 (12.69) years Female n = 56	N = 80	Clinical examination	**Thoracic pain prevalence** 54%
Sterling 2009 [59]	Cohort	**Acute WADI-III**	**WAD** Mean (SD) 36.27 (12.69) years Female n = 54	N = 85	Clinical examination	**BPTT (from 180-degree extension elbow, mean (SD))** • S-LANSS >12 n = 29–56.5 (28) • S-LANSS <12 n = 56–35.3 (19)
Wenzel 2009[60]	Case control	**Chronic WAD**(94% >2 years)	**WAD** Mean (SD) 47.6 (14.9) Female 48.4%	WAD N = 785	Questionnaire and clinical examination	**Control group** n = 2.060 (0.3%) **CWAD** n = 117 (41%) Odds ratio (95% confidence interval) 7.84 (6.26–9.82)
			**Control** Mean (SD) 47.9 (16.7) Female 50.7%	Control N = 52,208		
Wirth2014 [61]	Case control	**Chronic WAD**	WAD Mean (SD) age 45 (10.03) years Females n = 4 Males n = 3	N = 7 (sub set from larger cohort of neck pain patients)	Clinical examination	**Thoracic neutral posture and mobility** (raw data provided by authors) **WAD** • Thoracic neutral 30.14 (12.86) degrees • Thoracic flexion-extension 50 (14.55) degrees • Chest expansion axilla 4.72 (2.53) cm • Chest expansion xiphoid 5.21 (2.92) cm **Control** • Thoracic neutral 36.75 (11.32) degrees • Thoracic flexion-extension 43.76 (16.09) degrees • Chest expansion axilla 5.86 (2.34) • Chest expansion xiphoid 5.75 (2.33)
Yeung 1997 [62]	Case control	**WAD** <12mths(**suggestive WADI or II**)	**WAD** Mean 25.3 years Female n = 20	WAD N = 20	Clinical examination	**Stage1:** • WAD n = 12 (60%), • Control n = 2 (5%) (n = 8 of WAD group reported mid-thoracic symptoms) **Stage2:** WAD (90%), Control n = 26 (65%) reported symptoms (WAD n = 15 (75%), Control n = 23 (57.5%) reported mid-thoracic symptoms **Stage 3:** • Pain response in both groups mid-thoracic **Stage 4:** • WAD n = 17 (85%) reported mid-thoracic symptoms • Control n = 33 (82.5%) reported mid-thoracic symptoms **Stage 5:** Evidence of greater proportion of mid-thoracic pain reduction in WAD group compared to control. **Stages 6, 7:** • no data Mean (SD) ROM degrees • Control L 74 (3.82), R 74 (4.57) • WAD L 68 (3.3), R 68 (4.60)
			Control Mean 24.0 years Female n = 40	Control N = 40		

AIS: Abbreviated Injury Scale BPI: brachial plexus irritation, BPTT: brachial plexus tension test, CCFT: craniocervical flexion test, CT: computerised tomography, EMG: electromyography, MRI: magnetic resonance imaging, MTP: myofascial trigger point, NBPI: no brachial plexus irritation, NDI: Neck Disability Index, NRS: numerical rating scale, OR: odds ratio, RCT: randomised controlled trial, ROM: range of movement, SCM: sternocleidomastoid muscle, TOS: thoracic outlet syndrome, TDH: thoracic disc herniation, WAD: whiplash associated disorders, WDQ: Whiplash Disability Index

**Table 2:** Overview of included studies.

Author N = 37 papers	Measurement and summary outcome	Sample size n =	Acute/ sub acute	Chronic	WADI/II	WADII	WADII/III
	**Thoracic pain prevalence**						
Yeung 1997	75% (during slump)	20		x	x?		
Koelbeck 1999	No difference light touch (reduced PPT, hyperalgesia & referral)	11		x		x	
Bergland 2001	15.3%	242		x	x (minor)		
Hartling 2002	74%	380	x		x		x
Bock 2005	71% (allodynia)	22		x	x		
Sterling 2005	54%	***76		x			x
Holm 2007	Acute 52%, chronic 80%	56	x	x			
Wenzel 2009	41%	785		x			
Hincapie 2010	66–75%	6481	x				
Myran 2011	7–8.8%	46,895		x			
Bortsov 2014	47%	*948	x		x		
*Cornips 2014*	*Pain associated with herniated disc 4/10*	*10*		*x*			
McLean 2014	21% (upper thoracic/shoulder 23%)	711	x		x		
Johansson 2015	43%	3711	x	?			
	**Chest pain prevalence**						
Holm 2007	Acute 0%, chronic 6%	56	x	x			
Hincapie 2010*	19%	*6481*	x				
Myran 2011	6.9–7%	46,895		x			
Bortsov 2014*	19%	*948*	x		x		
McLean 2014*	8%	*948*	x		x		
	**Thoracic posture & mobility**						
Kall 2008	Flexion hypomobility	47	x		x		x
Helgadottir 2011a	No change	23		x		x	
Wirth 2014	Reduced kyphosis (chest expansion)	7		x			
	**Thoracic outlet syndrome prevalence**						
Capistrant 1976	86%	35		x			
Capistrant 1986	36%	111		x?			
Magnusson 1994	32%	38		x	x		x
Kai 2001	74%	110					
Alexandre 2005	Positive	24		x	x (mild)		
	**Brachial Plexus test**						
Ide 2001	Prevalence 38%	119	x	x			
Sterling 2002	Positive	156		x			x
Sterling 2003b	Positive	**80	x	x			x
Sterling 2004	Positive	80	x				x
*Omar 2007*	*Brachial plexus lesion*	*1*		*x*			*x*
Chien 2009	Positive	31		x		x	
Sterling 2009	Positive	85	x				x
Ferrari 2010	Positive	69	x		x		
	**Muscle activation**						
*Bodack 1998*	*Weak mid and lower trapezius*	*1*	*x*				*x*
Klein 2001	SCM muscle activation: normal	46		x		x (mainly)	
Sterling 2003a	SCM muscle activity heightened	**66	x				x
Sterling 2004	SCM muscle activity heightened	***80	x				x
Helgadottir 2011b	Serratus anterior activation: delayed onset and reduced duration of activation. No change for lower and mid trapezius	27		x		x	
	**Muscle pain and trigger points**						
Bismil 2005	Mid/Low trapezius enthesopathy (myofascial pain +TP) 48%	25		x		x	
Ettlin 2008	SCM and Scaleneus medius MTP prevalence 24%, 30%	47		x			
Fernandez-de-las-Perez 2012	SCM and Scaleneus medius MTP	20	x			x	
Castaldo 2014	SCM Latent and active TP	49		x	x (mild)		x

Note

*Same population

** same population

*** same population

SCM: sternocleidomastoid muscle

### Risk of bias within studies

Agreement of risk of bias following discussion was excellent with studies ranging considerably in quality from 1/9 to 7/9. Key methodological flaws included poor definition of cases, representativeness of cases, lack of non-exposed cohort, lack of blinding, controlling for risk factors beyond age and gender and clarity of follow up time point in cohort studies. There were many instances where information was missing and email requests for additional data or clarification was unforthcoming, resulting in ratings being ‘unclear’. See Table 3 for risk of bias for cohort studies and Table 4 for case control studies.

**Table 3:** Risk of bias assessment: Thoracic dysfunction in whiplash associated disorders: A systematic review and meta-analysis cohort studies.

Author	Dysfunction & sample size	Classification and time post injury Disability Pain	Summary results	Quality									Comments/study quality
				Selection ****				Comparability *		Exposure /Outcome ***			
				Representativeness of exposed cohort? (linked to WAD)	Selection of the non-exposed cohort e.g. drawn from same community as exposed cohort	Ascertainment of exposure (WAD)? E.g Secured records, structured interview	Demonstration that outcome of interest was not present at start of study?	Study controls for age/sex?	Study controls for at least 3 additional risk factors? E.g. previous history of neck pain, trauma	Assessment of outcome? E.g. Independent blind assessment, record linkage	Was follow-up long enough for outcome to occur?	Adequacy of follow-up of cohorts? E.g. Complete follow-up, or subjects lost to follow-up unlikely to introduce bias	
Alexandre 2005	Thoracic outlet syndrome N = 24	Mild Mean (range) 11 (2–48) mths	Brachial plexus entrapment due to TOS	No Sub group cervical spine injury included	No non exposed cohort	Yes Patient examination and ED documents	Yes Exclusion criteria provided	No	No	Yes Medical tests: NCS	Yes	Unclear	Mild, but with neurological deficits? Conservative management prior to examination Post injury period variable All patients offered surgery
Bismil 2012	Trapezius dysfunction n = 25	WADII>6mths, but not reported	Trapezius myofascial-entheseal dysfunction Enthesopathy (myofascial pain and trigger points)	Yes	No non exposedcohort	Yes Patient examination	No Not reported	No Not reported	No	No	Yes	Unclear	Clinical examination in medico legal practice Limited sample details Limited information on examination and outcome measures
Bock 2005	Thoracic allodynia N = 22	WADI & II3-18 mths	Thoracic allodynia evident in 70.97% (more in lower spinal segments, T8,9,10)	Yes	No non exposed cohort	Yes Recruitment from private ‘physiatry’/pain management practice	Yes Clear exclusion criteria	No Wide age range (wider with male population)	Unclear Exclusion criteria controlled other risk factors?	Yes	Yes No follow up?	No No follow up	Aim focus to reliability of measures
Bortsov 2014	Thoracic pain N = 948	WADI & II 6 weeks, 3,6,12 mths NRS6 weeks 3.8 (2.8) 6 months 3.1 (2.8) 1 year 3.2 (2.9)	Thoracic and chest pain prevalence at 6 weeks 47% and 19% respectively	Yes	No non exposed cohort	Yes ED department interview	Unclear No reference to prior msk injury	Yes Adjusted for age and sex	Unclear	Yes Range of approaches	Yes	Yes	Recruitment from ED Limited upper back data: included within axial data Selective reporting of results: timepoints, regions
Capistrant1976	Thoracic outlet syndrome N = 35	Unknown Acute: 3.5 mths (max 8mths) Chronic: mean 29mths (inc n = 1 16 years)	Thoracic outlet syndrome n = 30/35 (86%)	No With signs of TOS. Unclear re stage & severity	No non exposed cohort	Yes Private neurological practice.	No Not reported	No	No	Yes NCS records	Yes	Unclear	Private neurological practice Clarity re stage and WAD classification Inconsistent follow up periods Not accounted for other variables
Capistrant1986	Cervical Strain injuries N = 111 Of whom N = 40 was TOS	Unknown? Chronic 24month period	Thoracic outlet syndrome prevalence 36%	No Selected for presentation	No non exposed cohort	Yes Private neurological practice.	No Not reported	No	No	Yes NCS records	Yes	Unclear	Clarity re stage and WAD classification Variable follow up periods Not accounted for other variables Limited details of sample, follow up
Ferrari 2010	Brachial plexus N = 69	WADI & II <1 week and chronic (3 months) WDQ 64 (23) VAS 2.2	Brachial plexus provocation test	Yes Acute WAD	No non exposed cohort	Yes GP referral and assessment by specialist researcher	Yes Clear exclusion criteria	No	No	Unclear Researcher performed measurements (no blinding)	Yes	Yes 2 loss to follow up	Clear recruitment, reporting attrition and sample details. Prognostic study Incomplete data for all time points: baseline BPTT omitted
Hartling2002	Upper back pain N = 380	Sub-acute WADI-III	Upper back pain 74.6%	Yes From earlier paper	No non exposed cohort	Yes From ED	Yes From earlier paper	No	No	Unclear	Yes	Yes 87.9% follow up	Derivation of a clinical prediction rule study rather than cohort
Holm2007	Upper back pain N = 56	Acute-chronic WAD	Chest pain prevalence Acute 0% Chronic (6 weeks) 6% Thoracic pain prevalence Acute 52% Chronic 80%	No Claimants	No non exposed cohort	No Claimants	Unclear No information	Yes	No	Unclear Questionnaire	Yes	Yes 63% for all time points	Insurance claimants or filed claim
Hincapie2010	Thoracic pain N = 6481	WADI, II <30 days post traffic injury	Thoracic pain prevalence 65.5–75.3% Chest painprevalence18.9%	No Litigants	No non exposed cohort	Unclear Self-Report to insurance company	No	No Age 18yrs or older	No	Unclear Self-report/ pain drawings	Yes No follow up?	Yes No follow up?	Insurance claimants Analysed pain drawings linked to pre-existing symptoms- no data reported
Ide2001	Brachial plexus irritation N = 119	WAD1week to 6 months	Brachial plexus irritation prevalence n = 45/74 (61%) (associated with poor outcome)	Unclear	No non exposed cohort	Yes	No	No Was gender controlled? M = 59 vs W = 60	No	No	Yes	Yes 2 loss to follow up	Details of recruitment unclear Clarity re stage and WAD classification More females in BPI group Wide age range Assessor blinding unclear Loss to follow up unclear
Johansson2015	Thoracic pain N = 3711	WAD<42 days post injury when claim filed	Thoracic spine pain 43% Baseline pain rating 6 (5–8) 23% not recovered after one year	No Canadian provincial population	No non exposed cohort	Yes Registered health care professional	Unclear	Unclear Data provided for sub groups	No	Yes Independent research centre	Yes	Yes 90% follow up	Data for different time points not provided
Kai2001	Thoracic outlet syndrome N = 110	WAD	Neurogenic thoracic outlet syndrome prevalence n = 81/110 (74%)	Unclear Clarity re stage and WAD classification	No non exposed cohort	Yes	Unclear Not explicit re exclusion criteria	No	No	No	Unclear	Unclear	Details of recruitment unclear Sample details unclear Assessor blinding unclear Loss to follow up unclear Some had surgery
Kall2008	Cervico-thoracic mobility N = 47	WADI-III (sub-acute)	Concluded women with sub-acute WAD C7-T1 flexion hypomobility	Yes Self-selection	No non exposed cohort	Yes	Yes Clear eligibility criteria	No	Unclear	No	Yes	Unclear	Details of recruitment suggest self-selection into trial Sample details unclear
Magnusson1994	Thoracic outlet syndrome N = 38	Chronic WADI-III Mean (range) 17 (6–44) months	Thoracic outlet syndrome prevalence 31.6%	No Some had surgery	No non exposed cohort	Yes Interviewed by author	Yes Clear eligibility criteria	No	No	No	Yes	Unclear	Details of recruitment unclear Late whiplash Sample details unclear Some had surgery
McLean2014	Thoracic, chest pain N = 948	Acute WAD WADI & II 24 hours to 6 weeks	Thoracic pain prevalence 21%, shoulder 23%, chest 8%	No English speakers, non-Hispanic white population only	No non exposed cohort	Yes ED department interview	Unclear No reference to prior msk injury	Yes Adjusted for age and sex	Unclear	Yes	Yes	Yes	Non litigant population Clear eligibility criteria Only individuals from ED Payment for participation
Myran2011	Upper back and chest pain prevalence N = 46,895	WAD No other details	Chest/abdomen pain prevalence Men 7% Women6.9% Upper back pain prevalence Men 8.8% Women 7.0%	No Norwegian population	No non exposed cohort	Unclear Questionnaire as part of health survey	Unclear No reference to prior msk injury	Yes Adjusted for age and sex	Yes	Unclear	Yes	N/A	Non litigant population Clear eligibility criteria Only individuals from ED Payment for participation
Sterling 2005	Thoracic pain N = 76	WADII-III 6 months NDI 34.15 (2.37) VAS3.5 (1.2)	Thoracic pain prevalence 54%	Yes	No non exposed cohort	Yes Recruited primary care, ED and community	Yes Clear exclusion criteria	Yes	Yes Controls for other factors in analysis	Unclear	Yes	Yes Loss to follow up n = 4	Recruited primary care, ED and community Clear eligibility criteria Assessor blinding unclear Loss to follow up suggested 4/80, but numbers unclear
Sterling 2009	Brachial plexus N = 85	Acute WADII-III 2.6 (1.2) weeks	Brachial plexus provocation test positive (and associated with neuropathic pain presentation)	Yes Acute	No non exposed cohort	Yes Recruited primary care, ED and community	Yes Clear exclusion criteria	Unclear	Unclear	Unclear	Yes	Unclear	Recruited primary care, ED and community Assessor blinding unclear No mention of any losses to follow up. Control for age and gender unclear

**Table 4:** Risk of bias assessment: Thoracic dysfunction in whiplash associated disorders: A systematic review and meta-analysis case control studies.

Author	Dysfunction & sample size	Classification and time post injury Pain duration, disability and pain intensity	Summary results	Quality									Comments/study quality
				Selection ****				Comparability *		Exposure /Outcome ***			
				Is the case definitionadequate?	Representativeness ofcases?	Selection of controls? E.g. community controls	Definition of controls? E.g. no history of WAD or neck pain	Study controls for age/sex?	Study controls for at least 3 additional risk factors?	Assessment of outcome? E.g. Secure record, Structured interview byhealthcare practitioner, blind tocase/control status	Same method ofascertainment of cases/ controls?	Non-response rate? E.g. same for both groups	
Berglund 2001	Thoracic pain N = 242	Mild 7 years	Thoracic pain prevalence 15.3%	Unclear	No Litigant population	Yes	No ? prior injuries not accounted for	Yes Chosen based on age & sex	No	Yes Questionnaire	Yes	Yes ~77%	No patient demographics Funded by insurance company
Castaldo2014	Myofascial trigger points N = 49	WADII & III Mean (SD) 57.12 (14.11) mths VAS 5.59 (0.42)	SCM Myofascial trigger points (latent & active)	Yes Screen by physician	Yes	Unclear From same location as WAD	Yes	Yes Matched age & sex	Yes Gps comparable	Unclear	Yes	n/a	Compared with MNP Not clear re centre for recruitment Inc/exclusion criteria clear
Chien 2009	BPPT N = 31	WADII 16 (11) months post injury NDI 45.9 (18.8)	Brachial plexus provocation test positive in WAD	Unclear Primary care and media	Yes WADII	Yes Community	Yes Never experienced cervical pain or trauma	Yes Matched age & sex	No	Unclear No information	Yes	Unclear	Other factors not controlled for e.g. psychological factors WAD group moderate disability
Ettlin2008	Myofascial trigger points N = 47	Unknown Mean (SD) 1.47 (1.8) years VAS 5.42 (2.08)	Scaleneus medius and SCM myofascial trigger points	No	Unclear	Yes Same site	Unclear	No Similar age, but not gender	No	Yes Blinded assessor	Yes	Unclear	Controls recruited from one of the sites Unclear re sample characteristics More females in WAD group WAD participants had physiotherapy
Fernandez-Perez2012	Myofascial trigger points N = 20	WADII Mean (SD)26.6 (3.8) days NDI 68.5 (8.7) VAS 6.2 (2.6)	Scalene and SCM myofascial trigger points	Yes Referred by physician	Yes WADII severe disability	Yes General population	Unclear No reference to previous WAD	Yes Matched age & sex	Yes	Yes Blinded assessor	Yes	n/a	Other factors not controlled for e.g. psychological factors WAD group severe disability
Helgadottir2011a	Thoracic Alignment (mid thoracic curve) N = 23	WADII >6months NDI = 38 (18.75)	Thoracic alignment (posture) no change	No Self-selected	Yes WADII	No Convenience sample	Unclear Current history only	Yes	Yes	No Clear procedure	Yes	Unclear	VAS not provided although included Recruitment of control unclear
Helgadottir2011b	Muscle function (mid & lower trapezius, serratus anterior) N = 27	WADII >6monthsNDI>10 NDI 38 (18) VAS 6 (2)	Delayed onset and duration of serratus anterior muscle activation	Yes Physio screened	Yes WADII NDI>10	No Convenience sample	Unclear Current history only	Yes	Yes	No Clear procedure	Yes	Unclear	No reference to blinding VAS not provided Recruitment of control unclear
Klein2001	Muscle activation sternocleidomastoid N = 46	Chronic WADI-III 33.7 (26.7) months	No evidence of earlier SCM activation during axial rotation	Yes GP referral	Unclear No sure re classification	No Convenience sample	No Some controls had pre-existing neck complaints	Unclear Age adjusted for in analysis?	No	No Clear procedure	Yes	Unclear	Recruitment of controls unclear Controls younger than WAD WAD group 33% men, control 42%)
Koelbeck1999	Pain over infraspinatus region N = 11	Chronic WADII Mean (range) duration 4 years, 5 months (1 year, 1month to 7 years 5months) VAS 5.2 (0.9)	Reduced pressure pain threshold in WAD: muscle hyperalgesia and diffuse pain referral	Yes Referral to pain clinic	Yes WADII	No	Yes No history of musculoskeletal pain conditions	Yes Matched age & sex	No	No Clear procedure	Yes	Unclear	Recruitment from pain centre Control recruitment unclear
Sterling 2002	Brachial plexus N = 156	Chronic (>3mths) WAD II &III Unclear	Brachial plexus provocation test positive in WAD	Unclear Referral to research centre.	Yes WADII or III	Yes Community	Yes Never experienced cervical pain or trauma	No Gender imbalance, but analysis adjusted	No	No No information	Yes	Unclear	Time following injury unclear No reference to blinding Clinical examination done. No independent validation
Sterling 2003a	SCM activity N = 66	WADII & III1-3 months NDI from 3.00–47.9	SCM activity increased in those with mod/severe symptoms>mild>recovered and controls: over tome points to 3 months post injury	Unclear A&E, community, primary care. No reference to primary records	Yes WADII or III	Yes Community	Yes Never experienced cervical pain or trauma	Yes	Yes	No No information	Yes	Unclear No information provided	No reference to blinding Control for other psychological variables
Sterling 2003b	BPTT N = 80	Sub-acute WADII-III	BPTT: those with positive test within 1 mth of injury higher pain levels at 6 mths	Unclear A&E, community, primary care	Yes WADII or III	Yes Community	Yes Never experienced cervical pain or trauma	Yes	Yes	No No information	Yes	Unclear No information provided	No reference to blinding Control for other psychological variables
Sterling 2004	SCM activity and BPTT N = 80	Acute WADII & III<1 month NDI 15.6–69.5	SCM increased in WAD and with increasing symptom severity BPTT positive in WAD and with increased symptom severity	Unclear A&E, community, primary care	Yes WADII or III	Yes Community	Yes Never experienced cervical pain or trauma	Yes	Yes	Unclear Blind to subject responses from questionnaires	Yes	Unclear	Blinding unclear
Wenzel2009	N = 785	Chronic WAD >2 years	Thoracic spine pain prevalence 41%	No From a wider health study	Unclear	Yes Community	No Inc/exc not provided	Yes	No	Unclear Blind to subject responses from questionnaires	Yes	Unclear	Large sample as part of health study Controlling for other variables unclear
Wirth2014	Chest mobility, Thoracic flexion, extension N = 7	Chronic WAD 1–5+ years NDI 12 (7.1)	Reduced thoracic kyphosis & small reduction in chest expansion	No Recruited from sports centre	No	No	No Not reported	Yes Groups balanced	No	No No blinding	Yes	Unclear	Recruited from sports clubs and medical centres No information re blinding
Yeung 1997	Slump N = 20	WAD <12mths(suggestive WADI or II) Unclear	Slump test: positive in all WAD (mid-thoracic pain 75%)	No	No	No Unclear	Yes Clear exclusion criteria	Yes	No	No No blinding	Yes	Unclear	Only females Unclear recruitment for WAD and control Part of a reliability study

### Results of individual studies

From this review we identified evidence of the following dysfunction,: thoracic spine pain in acute/sub-acute/chronic WAD ranging from minor injuries to more severe (WADIII) presentations [11, 14, 17, 32, 33, 43, 50, 52, 58, 62]; chest pain in acute/sub-acute/chronic WAD [11, 17, 52, 53]; postural changes [44, 61] and reduced chest/thoracic mobility in CWAD [61]; thoracic outlet syndrome in CWAD [31, 35, 36, 47, 51]; involvement of the brachial plexus at all stages and across all levels of WAD severity [12, 46, 55, 57, 59]; muscle dysfunction in the form of the following: 1) heightened activity of the sternocleidomastoid during neck flexion [12, 56], 2) delayed onset of serratus anterior during arm elevation at the chronic stage in mild WAD [45] and 3) a high prevalence of myofascial pain and trigger points in the scalene muscles [40, 41], sternocleidomastoid [37, 40, 41] and mid/lower fibres of trapezius [16] within the sub-acute and chronic stages and across different levels of severity.

### Synthesis of results

Synthesis of findings from cohort and case control studies across outcomes of dysfunction are provided in Tables 5–8. Sub-grouping according to stages and defined populations are included where reported. A summary of overall quality is provided based on GRADE following appraisal of risk of bias, consistency, precision, directness and effect size. Clinical heterogeneity across samples with respect to severity of presentation, time post injury and outcomes precluded meta-analysis being performed. Few studies stated a primary aim which accurately reflected the aims and objectives of this review.

**Table 5:** Pain.

Outcome	Studies	Findings Prevalence % (95% confidence interval) unless stated	Studies	Overall GRADE
**Thoracic spine pain in acute/sub-acute WAD** N = 11,577	Bodack 1998	-Positive	6 cohort, 1 single case study	Very low—due to risk of bias, directness and precision
	Hartling 2002	-Upper back pain 74.6%		
	Holm 2007	-52%		
	Hincapie 2010	-Mid back 65.5 (64.4–66.7) -Upper thoracic 75.3 (74.3–76.4)		
	^a^ Bortsov 2014 (Mild)	-Mid back 47% (18% widespread, 29% localised)		
	^a^ McLean 2014 (WADI/II) (moderate or severe pain NRS4)	-Mid back 21% (range 18–24%) -Upper back 23% (range 20–26%)		
	Johansson 2015	-43%		
**Thoracic spine pain in chronic WAD** N = 48,117	Yeung 1997(?WADI/II)	-Slump	5 cohort, 3 case control, 1 case series	Very low—due to risk of bias, precision, consistency and directness
	Koelbeck 1999 (WADII)	-No different controls		
	Bergland 2001 (Minor)	-15.3% (10.4–21.5)		
	Bock 2005 (WADI/II)	-71% (allodynia)		
	Sterling 2005 (WADII/III)	-54%		
	Holm 2007	-4months 86%, -8 months 94%, -12 months 87%		
	Wenzel 2009	-41%		
	Myran 2011	-men 8.8% -women 7%		
	Cornips 2014	10 previously asymptomatic individuals significant pain		
**Thoracic spine pain in chronic WAD I/II** N = 655	Koelbeck 1999 (WADII)	-No different controls	3 cohort, 1 case control	Very low—due to risk of bias, precision, consistency and directness
	Bergland 2001 (Minor)	-15.3% (10.4–21.5)		
	Hartling 2002	-Upper back pain 74.6%		
	Bock 2005 (WADI/II)	-71% (allodynia)		
**Thoracic spine pain in chronic WADII/III** N = 76	Sterling 2005 (WADII/III)	-54%	1 cohort	Low??
**Chest pain in acute/sub-acute WAD** N = 7485	Holm 2007	-0%	4 Cohorts	Very low—due to risk of bias, precision, consistency and directness
	Hincapie 2010	-localised 0.05 (0.00–0.10) -widespread 18.9 (17.9–19.8)		
	aBortsov 2014 (mild)	-19% (10% localised -9% widespread)		
	aMcLean 2014 (WADI/II)	-8% (6–10%) (moderate or severe pain)		
**Chest pain in chronic WAD** N = 46,951	Holm 2007	-6-weeks 6% -4-months 4% 8-months 12%, -12-months 22%	2 cohorts	Very low—due to risk of bias, precision, directness
	Myran 2011	-men 7%, women 6.9%		

**Table 6:** Posture and mobility.

Outcome	Studies	Findings	Grade	Overall GRADE
**Thoracic posture in chronic WAD** N = 30	Helgadottir 2011a (WADII)	-No change	2 Case control	Very low: due to risk of bias, precision, consistency and directness
	Wirth 2014	-Reduced kyphosis		
**Thoracic and chest mobility in chronic WAD** N = 54	Kall 2008	-Flexion hypomobility in women	1 cohort study,1 case control	Very low: due to risk of bias, precision, consistency and directness
	Wirth 2014	-Reduced chest mobility -Slight increase in thoracic mobility		

**Table 7:** Thoracic outlet syndrome and brachial plexus.

Outcome	Studies	Findings [TOS: Prevalence % (95% confidence interval); Brachial plexus provocation test (BPPT) unless stated]	Grade	Overall GRADE
**Thoracic outlet syndrome in chronic WAD** **N = 318**	Capistrant 1976	-86%	5 Cohort studies	Very low: due to risk of bias, directness, precision, reporting bias
	Capistrant 1986	-36%		
	Magnusson 1994	-31.6%		
	Kai 2001	74%		
	Alexandre 2005	-Positive secondary to BPI		
**Brachial Plexus in all WAD** **(n = 620)**	Ide 2001	BPI 38%	4 case control,3 cohort	Very low: due to risk or bias, directness and precision
	Sterling 2002 (WADII/III) Chronic	-WAD -26.21 (95%CI -28.05, -24.37), VAS 4.93 (4.6, 5.3) -Control -12.92 (95%CI -15.24, -10.6), VAS 2.62 (2.2, 3.04)		
	Sterling 2003b (WADII/III) Acute/sub-acute	**<1 months post injury [mean (SD)** • Recovered group -23.95 (2.4) degrees, VAS 1.8 (04) • Mild pain -33.97 (2.6) degrees, VAS 3.2 (0.5) • Moderate/severe pain -34.27 (3.4) degrees, VAS 4.1 (0.5) • Control -20.67 (3.12) degrees, VAS 1.8 (04) Mild pain recovered and no different from controls at 2 months		
	Sterling 2003b (WADII/III) Chronic	**6 months** Moderate/severe pain: continued to have higher VAS 3.4 (1.0) and reduced elbow extension (no data provided)		
	Sterling 2004 (WADII/III) Acute/sub-acute	**Mean (SD) degrees/VAS** • Mild symptoms -26.7 (17.7), VAS 1.8 (1.7) • Moderate symptoms -31.3 (14.9), VAS 3.0 (1.8) • Severe symptoms -36.5 (11.8), VAS 4.3 (1.6) • Control -21.4 (10.8), VAS 1.7 (1.7)		
	Sterling 2009 (WADI-III) Acute	**Mean (SD)degrees** • S-LANSS >12–56.5 (28) • S-LANSS <12–35.3 (19)		
	Chien 2009 (WADII)	WAD -22.3 (27.4) degrees VAS 2.4 (2.3) Control -11 (5.9) VAS 0.7 (1.1)		
	Ferrari, 2010 (WADI/II	**3 months:** • VAS: mean (SD)2.2 (1.2) • Elbow extension: 41.5 (23) degrees		
**Brachial Plexus In acute/sub-acute WAD** (n = 419)	Ide 2001	BPI 38%	2 case control,4 cohort	Very low: due to risk or bias, directness, precision
	Sterling 2003b (WADII/III)	**<1 months post injury [mean (SD)** • Recovered group (NDI<8) -23.95 (2.4) degrees, VAS 1.8 (04) • Mild pain (NDI 10–28) -33.97 (2.6) degrees, VAS 3.2 (0.5) • Moderate/severe (NDI >30) -34.27 (3.4) degrees, VAS 4.1 (0.5) • Control -20.67 (3.12) degrees, VAS 1.8 (04)		
	Sterling 2004 (WADII/III)	**Mean (SD)degrees/VAS** (pain & disability) • Mild (NDI 15.6) -26.7 (17.7), VAS 1.8 (1.7) • Moderate (NDI 39.5) -31.3 (14.9), VAS 3.0 (1.8) • Severe (NDI 69.5)-36.5 (11.8), VAS 4.3 (1.6) • Control -21.4 (10.8), VAS 1.7 (1.7)		
	Sterling 2009 (WADI-III)	Mean (SD)degrees • S-LANSS >12–56.5 (28) • S-LANSS <12–35.3 (19)		
	Ferrari, 2010 (WADI/II)	**3 months:** • VAS: mean (SD)2.2 (1.2) • Elbow extension: 41.5 (23) degrees		
**Brachial Plexus In chronic WAD** (n = 432)	Sterling 2002 (WADII/III)	-WAD -26.21 (95%CI -28.05, -24.37), VAS 4.93 (4.6, 5.3) -Control -12.92 (95%CI -15.24, -10.6), VAS 2.62 (2.2, 3.04) -Mild (NDI 10–28): recovered and no different from controls at 2 months	4 case control, 2 cohort	Very low: due to risk or bias, directness, precision
	Sterling 2003b (WADII/III)	**6 months** Moderate/severe (NDI >30): continued to have higher VAS 3.4 (1.0) and reduced elbow extension (no data provided)		
	Sterling 2004 (WADII/III)	**Mean (SD)degrees/VAS** (pain & disability) • Mild (NDI 15.6) -26.7 (17.7), VAS 1.8 (1.7) • Moderate (NDI 39.5) -31.3 (14.9), VAS 3.0 (1.8) • Severe (NDI 69.5)-36.5 (11.8), VAS 4.3 (1.6) • Control -21.4 (10.8), VAS 1.7 (1.7)		
	Chien 2009 (WADII)	WAD -22.3 (27.4) degrees VAS 2.4 (2.3) Control -11 (5.9) VAS 0.7 (1.1)		
	Sterling 2009 (WADI-III)	**Mean (SD) degrees** • S-LANSS >12–56.5 (28) • S-LANSS <12–35.3 (19)		
**Brachial Plexus In WADII/III** (n = 416)	Sterling 2002 (WADII/III) Chronic	WAD -26.21 (95%CI -28.05, -24.37), VAS 4.93 (4.6, 5.3) Control -12.92 (95%CI -15.24, -10.6), VAS 2.62 (2.2, 3.04)	4 case control	Very low: due to risk or bias, directness
	Sterling 2003b (WADII/III) acute/sub-acute	**<1 months post injury [ROM mean (SD)** (classified per pain & disability) • Recovered group (NDI<8) -23.95 (2.4) degrees, VAS 1.8 (04) • Mild pain (NDI 10–28) -33.97 (2.6) degrees, VAS 3.2 (0.5) • Moderate/severe (NDI >30) -34.27 (3.4) degrees, VAS 4.1 (0.5) • Control -20.67 (3.12) degrees, VAS 1.8 (04) Mild (NDI 10–28):recovered and no different from controls at 2 months		
	Sterling 2003b (WADII/III) Chronic	**6 months** Moderate/severe (NDI>30): continued to have higher VAS 3.4 (1.0) and reduced elbow extension (no data provided)		
	Sterling 2004 (WADII/III) Acute/sub-acute	**Mean (SD)degrees/VAS** (pain & disability) • Mild (NDI 15.6) -26.7 (17.7), VAS 1.8 (1.7) • Moderate (NDI 39.5) -31.3 (14.9), VAS 3.0 (1.8) • Severe (NDI 69.5)-36.5 (11.8), VAS 4.3 (1.6) • Control -21.4 (10.8), VAS 1.7 (1.7)		
	Chien 2009 (WADII)	WAD -22.3 (27.4) degrees, VAS 2.4 (2.3) Control -11 (5.9), VAS 0.7 (1.1)		

BPI: brachial plexus irritation, BPPT: brachial plexus provocation test, NDI: Neck Disability Index, VAS: visual analogue scale, ROM: range of movement, S-LLANS: short version Leeds Assessment of Neuropathic Symptoms and Signs

**Table 8:** Muscle dysfunction (muscle activation, pain and trigger points).

Outcome	Studies	Findings Percentage (SD) change in activation (EMG) unless stated	Grade	Overall GRADE
**Muscle activation: all muscles-** (n = 220)	Klein 2001	-no change	4 case control, 1 single case study	Very low—due to risk or bias, precision, consistency, directness
	Sterling 2003a	SCM (CCFT) • Recovered 29(4), • Mild 27(3), • Moderate/severe 40(4), • Control 16(3)		
	Sterling 2004 (acute)	SCM (CCFT) • Control 13(3), • Mild 32 (3), • Mod 29(4); • Severe 47(4)		
	Omar 2007 (chronic WADIII)	-Weak mid/lower trapezius		
	Helgadottir 2011b	-serratus anterior delayed onset, mid/ low trapezius unchanged		
**Muscle activation: all muscle- Acute/sub-acute**(n = 146)	Sterling 2003a	SCM (CCFT) • Recovered 29 (4) • Mild 27 (3) • Moderate/severe 40 (4) Control 16 (3)	2 case control	Very low–due to risk of bias
	Sterling 2004	- SCM (CCFT) Control 13 (3), Mild 32 (3), Mod 29 (4), Severe 47 (4)		
**Muscle activation: all muscles- Chronic WAD**(n = 73)	Klein 2001	-no change	2 case control, 1 single case study	Very low—due to risk or bias, precision, consistency and directness
	Omar 2007	-Weak mid/lower trapezius		
	Helgadottir 2011b	-serratus anterior delayed onset, mid and lower trapezius unchanged		
**Muscle activation: SCM (All stages)** (n = 192)	Klein 2001	-no change	3 case control	Very low—due to risk or bias, precision, consistency and directness
	Sterling 2003a	SCM (CCFT) • Recovered 29 (4), • Mild 27 (3), • Moderate/severe 40 (4)		
	Sterling 2004	SCM (CCFT) • Control 13 (3), • Mild 32 (3), • Mod 29 (4), • Severe 47 (4)		
**Muscle activation: SCM- Sub-acute WAD II/III** (n = 66)	Sterling 2003a	SCM (CCFT) • Recovered 29 (4) • Mild 27 (3) • Moderate/severe 40 (4) • Control 16 (3)	1 case control	Very low–due to risk of bias
Muscle activation: SCM-Chronic WAD II/III (n = 126)	Klein 2001	-no change	2 case control	Very low—due to risk or bias, precision, consistency
	Sterling 2004	SCM (CCFT) • Control 13 (3) • Mild 32 (3) • Mod 29 (4) • Severe 47 (4)		
**Myofascial pain and trigger points: all muscles -Sub-acute WADII** (n = 20)	Fernandez-de-las-Perez 2012	**Number of TPs** • **WAD Scalene:** Active R6, L4; Latent R9, L10; No R5, L6 • **Control Scalene:** Active R0, L0; Latent R2, L4; No R18, L16 • **WAD SCM:** Active R1, L6; Latent R12, L9; No R7, L5 • **Control SCM:** Active R0, L0; Latent R4, L5; No R6, L15	1 case control	Very low—due to risk or bias
**Myofascial pain and trigger points: all muscles-Chronic WAD**(n = 121)	Bismil 2005 (WADII)	**Prevalence:** 48%	2 case control, 1 cohort	Very low—due to risk or bias, reporting bias, directness
	Ettlin 2008	**Prevalence:** • **WAD:** Scaleneus medius 63.8%, SCM 51.1% • **Control:** Scaleneus medius 12.5%, SCM 12.5%		
	Castaldo 2014 (WADII & III)	**Number of TPs (SCM)** • **WAD:** Active L 21, R 22; Latent L 13, R 15 • **Control** (MNP): Active L 13, R 13; Latent L 6, R 9		
**Myofascial pain: Trapezius (mid/low)** **Chronic WADII** (n = 25)	Bismil 2005	Prevalence: 48%	1 cohort	Very low—due to risk or bias, precision, reporting bias, directness
**Myofascial pain and trigger points: SCM-** **Chronic and sub-acute WAD** (n = 116)	Ettlin 2008	**Prevalence** • **WAD:** 51.1% • **Control:** 12.5%	3 case control	Very low—due to risk or bias, precision (?), reporting bias, directness
	Fernandez-de-las-Perez 2012	**Number of TPs** • **WAD:** Active R1, L6; Latent R12, L9; No R7, L5 • **Control:** Active R0, L0; Latent R4, L5; No R 6, L15		
	Castaldo 2014	**Number of TPs** • **WAD:** Active L 21, R 22; Latent L 13, R 15 • **Control (MNP):** Active L13, R 13; Latent MTP L 6, R 9		
**Myofascial pain and trigger points: Scalene-** **Chronic WAD and sub-acute** (n = 67)	Ettlin 2008	**Prevalence** • WAD: 63.8% • Control: 12.5%	2 case control	Very low—due to risk or bias, precision, directness
	Fernandez-de-las-Perez 2012	**Number of TPs** • **WAD**: Active R 6, L4; Latent R 9, L10; No R 5, L6 • **Control**: Active R0, L0; Latent R2, L4; No R 18, L16		

SCM: sternocleidomastoid, CCFT: craniocervical flexion test, MTP: myofascial trigger points, MNP: mechanical neck pain

#### Thoracic spine pain

Despite the very low quality of included studies, there is evidence of thoracic spine pain in a sub-acute WAD population (n = 11,576) [11, 14, 17, 34, 39, 43, 52, 60], with prevalence ranging between 21%-66%,. Findings were inconsistent in chronic WAD, with prevalence ranging 0–94% [17, 50]. Study quality, differing time points post injury, differing measurement approaches and higher degrees of WAD severity could partly account for the inconsistency. For example, chronic WAD, studies with less severe presentations (minor or WADII) [32, 50] demonstrated lower prevalence rates (0–15.3%) compared to studies investigating WADII/III where prevalence was 54% [58]. Just one study reporting thoracic pain specifically associated with central sensitisation (allodynia) where pain prevalence was 71% in individuals with CWADI/II [33].

#### Chest pain

Prevalence of chest pain in acute/sub-acute was reported to range 0.0–19% [11, 14, 17, 52], although when considered as part of a widespread pain presentation ranged 9–19% [11, 14]. In one study where they only considered individuals with numerical rating scale (NRS 0–10) 4+ chest pain prevalence was lower at 8% [52]. In CWAD prevalence rates for chest pain ranged 6–22% [17, 53] although this reflects a, broad timescale with one study reporting results 6 month post injury [17] to one exploring prevalence at any time point following injury [53]. Drawing definitive conclusions on prevalence of chest pain is difficult given variation in approaches used to record pain (pain drawings, VAS, *etc*.), time points post injury, sample heterogeneity and the overall methodological low quality of research. This may also be a related to the focus of this review, being to those with mild to moderate presentations of WAD where studies including WADIV or fractures were excluded.

#### Thoracic posture

Evidence of thoracic postural dysfunction is inconclusive given conflicting findings from a small number of studies of low methodological quality evidence where postural assessment was not a primary focus [44, 61]. Future studies should consider the use of a gold standard measure for postural evaluation [63].

#### Thoracic mobility and chest mobility

There is a notable gap in the evidence exploring thoracic and chest mobility in WAD, with just two studies (n = 54) of very low quality suggesting a trend for reduced chest mobility (p>0.05) and flexion hypomobility at the cervico-thoracic junction in women. In terms of thoracic mobility, evidence from one study (n = 7) [61] suggests a slight increase in thoracic flexion-extension, although with such a small sample and lack of inclusion of a valid approach to quantify thoracic mobility, meaningful conclusions cannot drawn.

#### Thoracic outlet syndrome in CWAD

Five studies found, a prevalence of 31–74+% [31, 35, 36, 47, 51] of thoracic outlet syndrome in CWAD. Although this suggests a relatively high prevalence, the methodological quality of included studies was generally poor (<4/9), resulting in a very low rating of quality according to GRADE.

#### Brachial plexus

**Acute/sub-acute WAD**

Notwithstanding that evidence is drawn principally from one research group, there is evidence of thoracic dysfunction in relation to the brachial plexus provocation test (BPPT) in WADI-III. From the research by Sterling et al [12, 57, 59], there is evidence of a negative association between self-report symptom severity (VAS) and range of elbow extension during the BPPT in chronic WAD. Moreover, the extent of this dysfunction is in turn related to the degree of pain and disability, with those participants presenting with higher levels of self-reported pain and disability (NDI) having greater levels of dysfunction during BPPT.

**Chronic WAD**

For participants with CWADII and/or III, evidence indicates dysfunction detected during the BPPT, with pain levels and restriction in elbow extension almost double those found in asymptomatic controls [38, 55]. Furthermore, those with higher levels of self-reported pain and disability (NDI>30) continued to have evidence of dysfunction 6 months after the injury which was not seen in the mild group who were no different to the recovered group at 2 months [57]. Although quality of individual studies varied (4-6/9), the overall body of evidence for dysfunction of the brachial plexus remains very low overall, primarily due to risk of bias of the included studies.

#### Muscle activation

There are limited and very variable findings of thoracic muscle dysfunction (activation) in WAD with studies investigating a relatively small number of muscles: sternocleidomastoid [12, 49, 56]; serratus anterior [45]; middle and lower fibres of trapezius [45, 54]. Although it is difficult to derive meaningful conclusions with respect to serratus anterior and trapezius, there is evidence supporting changes in sternocleidomastoid muscle activation with heightened levels of activation during a task of cranio-cervical flexion; this increase in sternocleidomastoid activity however, was not seen during neck rotation [49]. There appears to be a positive relationship between sternocleidomastoid activation and higher levels of pain severity, with participants with CWADII/III and moderate to severe levels of disability demonstrating increased levels of sternocleidomastoid activation of between 27–47% [12, 56]. Individuals with mild, moderate/severe presentations all share comparative levels of sternocleidomastoid activation in the acute [56] and chronic phases [12].

#### Myofascial pain and trigger points

Myofascial trigger points are highly prevalent in WAD with estimates ranging between 48–64% [16, 37, 40]. Muscles that have been investigated include the middle/lower trapezius [16], scaleneus medius [40, 41] and sternocleidomastoid [37, 40, 41], all with similar prevalence levels. Findings suggest that latent trigger points are more prevalent in sub-acute WADII [41] whereas in chronic WAD there is a higher prevalence of active trigger points [37]. It should however be noted that the sample of chronic WAD comprised both WADII and III, so perhaps severity could partly explain the differences seen. Likewise, age may account for some of the differences given the sub-acute sample was 28.7 years [41] compared 41.6 years in the chronic WAD group [37].

## Discussion

### Summary of evidence

This is the first methodologically rigorous systematic review investigating thoracic dysfunction in whiplash associated disorders. From a comprehensive search, 38 studies were included and evaluated as part of the review. Many studies were at risk of bias, primarily due to poor reporting with most studies published prior to the introduction of the STROBE reporting guidelines for observational studies. Notwithstanding the low quality of the evidence, there is unequivocal evidence of thoracic dysfunction in WAD.

#### Pain

Although there is a high prevalence of acute and chronic pain experienced in the thoracic spine region following injury, distinguishing the interplay of pain mechanisms is however challenging. Whilst we know peripheral and central sensitisation begin immediately following injury [64], it is plausible that damage to thoracic musculoskeletal tissues contributes to the relatively high prevalence of pain reported in the acute/sub-acute stages from peripheral nociceptor stimuli [11, 14, 17, 43, 52]. Relatively few studies in this review reported perceived pain levels e.g. VAS, making it difficult to consider an association between injury severity and tissue damage. Although reviewed in detail by Van Oosterwijck et al, [64] the current review found just one study reporting thoracic pain specifically associated with central sensitisation, with allodynia reported in 71% of individuals with CWADI/II [33], arguably contributing to the lack of consistency with findings for chronic WAD where differing pain mechanisms may co-exist. Likewise distinguishing local from referred pain is challenging where injury in the cervical spine may refer pain caudally to the upper and mid thoracic region [65]. Unlike the thoracic spine, reported chest pain prevalence was relatively low, perhaps more closely associated with severe injuries, including fractures, which were excluded from this review. Future studies should, in addition to using the QTF Classification, include self-reported pain severity for each anatomical region.

#### Thoracic posture and mobility

With limited very low quality evidence and relative to the cervical spine, there is a paucity of research investigating thoracic posture and mobility in WAD [44, 45, 48, 61]. This may be explained with priority being given to areas with most severe pain, with the cervical spine and associated tissues being most vulnerable to stress and damage compared to the relatively stable and stiff thoracic spine [21], arguably enhanced with the mandatory use of seatbelts offering additional stability. However, considering the effect of a forceful injury on posterior structures, and neck stiffness being a hallmark of chronic WAD further research is required, specifically to investigate thoracic posture, mobility and muscle stiffness in WAD, all of which may offer new directions for research into management of WAD.

#### Thoracic outlet syndrome

Although this review suggests a relatively high prevalence of thoracic outlet syndrome, the quality of evidence is very low and derived from relatively older studies, including two studies from the same group of authors [35, 36]. Whilst the mechanism of injury and resultant strain on the scalene triangle in WAD would, in theory, place the thoracic outlet at risk of injury, this does needs to be investigated further. Likewise as a ‘syndrome’, this does not provide primary evidence of a primary structural dysfunction; it merely provides evidence of a dysfunction which, in turn, could be neurogenic, myogenic, vasculogenic in nature. Perhaps, in the absence of evidence of thoracic outlet syndrome in acute WAD, this condition is secondary to the consequences of whiplash, altered posture, changes in muscle behaviour *etc*. [66]. Future studies should use robust observational study designs and include valid assessment techniques to diagnose thoracic outlet syndrome.

#### Brachial plexus

There is considerable evidence of brachial plexus dysfunction in both acute/sub-acute and chronic WAD from research investigating the brachial plexus provocation test. Although coming from a relatively small group of researchers, the evidence supports further investigation. Future research could usefully consider the relationship of brachial plexus dysfunction to other musculoskeletal changes in the cervical and thoracic spine following a whiplash injury, but also approaches to managing this; a notable gap within the whiplash management evidence [9, 10, 66]. Although inclusion of the brachial plexus in this review may at first appear tenuous, with contributions from the level of T1 and its relationship to the thoracic outlet, its inclusion provides good evidence to further explore this anatomically and functionally challenging cervico-thoracic-supraclavicular region; a transitional zone between the stable/stiff thoracic spine and relatively mobile cervical spine.

#### Muscle activation

There is unequivocal evidence of altered function of muscles following whiplash injury [66], however this evidence is largely limited to cervical muscles, with this review identifying just three muscles with insertions to the thoracic region, sternocleidomastoid [12, 49, 56]; serratus anterior [45]; middle and lower fibres of trapezius [45, 54]. Although it is difficult to derive meaningful conclusions with so few muscles investigated and the quality of the evidence, the observed positive relationship between sternocleidomastoid activation and pain severity, and evidence across all stages post injury [12, 49, 56] supports the need for further research into altered activation of cervico-thoracic and thoracic muscles, ideally involving functional spinal movements; something that is now feasible with advances in technology, including high density EMG.

#### Myofascial pain and trigger points

This review has identified that myofascial trigger points are highly prevalent in WAD [16, 37, 40], although again from very low quality evidence and limited to a small number of muscles; middle/lower trapezius [16], scaleneus medius [40, 41] and sternocleidomastoid [37, 40, 41]. Nonetheless all muscles have similar prevalence levels of trigger points, with a higher prevalence of latent trigger points in sub-acute WAD [41] and active trigger points in chronic WAD [37]. With evidence of trigger points across all muscles and across the stages and severity of presentations, research is now required to explore other muscles in the thoracic region and better understand the development of pain, and persistent pain seen in chronic WAD, perhaps with longitudinal studies. Notwithstanding the quality, evidence was found of myofascial-entheseal dysfunction [16], a relatively new clinical entity and arguably similar to insertional tendinopathies. With rapid advances in our understanding of injury induced tendinopathies in the lower limb, this does offer new insights to possible muscle pathologies which may contribute to persistent pain and disability seen in WAD.

This review provides unequivocal evidence of thoracic dysfunction in WAD, albeit from evidence of low/very low quality. The findings do support a more extensive clinical evaluation of patients following a whiplash injury and the need for more methodologically robust observational studies to further characterise thoracic dysfunction in WAD across stages of the condition and levels of severity. Knowledge and understanding of thoracic dysfunction, where anatomical and biomechanical relationships with the cervical spine exist, offers novel directions for research into management of this disabling condition.

Research into WAD management has been, and continues to be primarily focused on managing cervical spine dysfunction, with interventions such as manual therapy, exercise *etc*. targeting the cervical spine [66], and targeting the psychological impact of a whiplash injury [66]. With inconclusive evidence of the therapeutic value of the above [67] it is perhaps time to consider new directions for research.

Whilst exercise as part of multimodal packages of care is recommended in the management of acute and chronic WAD [66, 68], the range of approaches available are considerable e.g. graded functional exercise, postural exercises, and strengthening and motor control exercises *etc*.[66]. The authors are not however aware of any recommendations or research specifically supporting the inclusion of thoracic spine exercises, although these could reasonably be incorporated within functional exercise programmes. With at best short term modest improvements in current exercise interventions [9, 10] and some evidence of some therapeutic value of thoracic spine manipulation in WAD [69] further research to investigate interventions targeting the thoracic spine and related dysfunction is justified.

It has not until recent years that the thoracic spine, coined the ‘Cinderella’ of the spine [21], has started to receive more research interest. This has been attributed partly to the relatively lower prevalence of symptoms than the cervical and lumbar spine more generally, but also due to the lack of affordable, non-invasive and valid measurement tools to evaluate motion in this anatomically complex and relatively stiff spinal region [21]. With the development of new measurement approaches for thoracic posture and mobility [63, 70, 71] we now have tools to support research of thoracic posture and mobility in the thoracic spine region in painful neck disorders.

### Strengths and limitations of this review

This review is rigorous and original, with a design and focus on the thoracic spine region using a pre-defined rigorous and published protocol with subject and methodological experts contributing to the evaluation. The key limitation of the review is lack of high quality evidence and compounding this was that few authors responded to requests for additional information or data.

## Conclusions

This first and rigorous systematic review found considerable evidence of thoracic pain and dysfunction in patients at all stages following whiplash injury. Notwithstanding the low/very low level of evidence, our findings do support a more extensive clinical evaluation of patients presenting with WAD. Key findings include 1) a high prevalence of thoracic spine pain, with the highest levels of pain immediately following injury and in more severe presentations 2) evidence of muscle dysfunction (delayed onset or heightened levels of activity) in a limited number of muscles 3) evidence of thoracic outlet syndrome and brachial plexus involvement 4) inconclusive/limited evidence of postural changes and effect on thoracic spinal mobility. Additional high quality research is required to further characterise dysfunction across other structures in the thoracic region, including but not limited to the thoracic spine (mobility and posture) and thoracic muscles (stiffness, activation patterns). In turn this may inform the design of clinical trials targeting such dysfunction.

## Supporting information

S1 Table PRISMA 2009 checklist. (DOC)

S2 Table Medline search. (DOCX)

S3 Table Excluded papers and reasons. (DOCX)
