# Supplementary material for: Thoracic dysfunction in whiplash associated disorders: A systematic review
Source: PLoS One. 2018 Mar 23;13(3):e0194235. doi: 10.1371/journal.pone.0194235 (PMC5865734; doi:10.1371/journal.pone.0194235)
Supplement: S3 Table — (DOCX) [file pone.0194235.s003.docx]

Additional file 3: Excluded studies and reasons

| Ansari et al 2001 (1) | Case studies recording fractures |
| --- | --- |
| Banic et al 2004 (2) | Upper trapezius (no response from authors) |
| Bartsch et al 2008 (3) | Injury analysis |
| Buskila, 1997 (4) | fibromyalgia |
| Crotti, 2005 (5) | Review |
| Doud et al, 2015 (6) | No pain or dysfunction data |
| Elert et al, 2001 (7) | Upper trapezius only |
| Ferrantelli, 2005 (8) | Included fracture |
| Fernandez-de-las-Penas and Cleland, 2005 (9) | Letter to editor |
| Ferrari, 2002 (10) | Letter |
| Gerdle et al, 2008a (11) | Upper trapezius only |
| Gerdle et al, 2008b (12) | Upper trapezius only |
| Helgadottir et al, 2010 (13) | Scapular |
| Inaba et al, 2015 (14) | Thoracolumbar blunt trauma |
| Iumashev , 1970 (15) | Simulation study |
| Kosek, 2008 (16) | Infraspinatus |
| Langdorf , 2014 (17) | Blunt trauma and not compatible focus |
| Lemming et al, 2012 (18) | No thoracic specific data |
| Moog et al, 2002 (19) | Sternum: requested data: no response |
| Nirula 2008 (20) | No thoracic specific data |
| Raak & Wallin, 2006(21) | Upper trapezius. No data for thoracic region. |
| Rao et al, 2014 (22) | Fractures |
| Rotstein , 1986 (23) | Visceral injury |
| Sjostrom et al, 2003 (24) | Sway |
| Wallin et al, 2008 (25) | Does not specify UT or mid traps. |
| Waxman, 1996 (26) | Review |

1. Ansari SA, Mandoorah M, Abdalrahim M, Al Moutaery KR. Dorsal spine injuries in Saudi Arabia - An unusual cause. Surgical Neurology. 2001;56(3):181-4.

2. Banic B, Petersen-Felix S, Andersen O, Radanov B, Villiger P, Arendt-Nielsen L, et al. Evidence for spinal cord hypersensitivity in chronic pain after whiplash injury and in fibromyalgia. Pain. 2004;107(1-2):7-15.

3. Bartsch AJ, Gilbertson LG, Prakash V, Morr DR, Wiechel JF. Minor crashes and 'whiplash' in the United States. Annals of advances in automotive medicine / Annual Scientific Conference Association for the Advancement of Automotive Medicine Association for the Advancement of Automotive Medicine Scientific Conference. 2008;52:117-28.

4. Buskila. Increased rates of Fibromyalgia following Cervical spine injury. 1997.

5. Crotti FM CA, Carai M, Sgaramella E, Sias W. Post-traumatic thoracic outlet syndrome (TOS). Acta Neurochir Suppl. 2005(92):13-5.

6. Doud AN, Weaver AA, Talton JW, Barnard RT, Meredith JW, Stitzel JD, et al. Has the incidence of thoracolumbar spine injuries increased in the United States from 1998 to 2011? Clinical Orthopaedics & Related Research. 2015;473(1):297-304.

7. Elert J, Kendall SA, Larsson B, Mansson B, Gerdle B. Chronic pain and difficulty in relaxing postural muscles in patients with fibromyalgia and chronic whiplash associated disorders. The Journal of rheumatology. 2001;28(6):1361-8.

8. Ferrantelli JR HD, Harrison DD, Stewart D. Conservative treatment of a patient with previously unresponsive whiplash-associated disorders using clinical biomechanics of posture rehabilitation methods. Journal of manipulative and physiological therapeutics. 2005;28(3):e1-8.

9. Fernandez-de-las-Penas C, Cleland JA. Management of whiplash-associated disorder addressing thoracic and cervical spine impairments: a case report. The Journal of orthopaedic and sports physical therapy. 2005;35(3):180-1.

10. Ferrari R BT, Wilbourn AJ. Thoracic outlet syndrome (TOS) is one of the traumatic complications of whiplash injury. Journal of spinal disorders & techniques. 2002;15(4):334-5.

11. Gerdle B, Hilgenfeldt U, Larsson B, Kristiansen J, Sogaard K, Rosendal L. Bradykinin and kallidin levels in the trapezius muscle in patients with work-related trapezius myalgia, in patients with whiplash associated pain, and in healthy controls - A microdialysis study of women. Pain. 2008;139(3):578-87.

12. Gerdle B, Lemming D, Kristiansen J, Larsson B, Peolsson M, Rosendal L. Biochemical alterations in the trapezius muscle of patients with chronic whiplash associated disorders (WAD)--a microdialysis study. Eur J Pain. 2008;12(1):82-93.

13. Helgadottir H, Kristjansson E, Mottram S, Karduna AR, Jonsson H, Jr. Altered scapular orientation during arm elevation in patients with insidious onset neck pain and whiplash-associated disorder. The Journal of orthopaedic and sports physical therapy. 2010;40(12):784-91.

14. Inaba K, Nosanov L, Menaker J, Bosarge P, Williams L, Turay D, et al. Prospective derivation of a clinical decision rule for thoracolumbar spine evaluation after blunt trauma: An American Association for the Surgery of Trauma Multi-Institutional Trials Group Study. The Journal of Trauma and Acute Care Surgery. 2015;78(3):459-65; discussion 65-7.

15. Iumashev GS GA, Dmitriev AE, Pyrlina NP, Antuf'ev II. Experimental anc clinical whiplash injuries of the cervical and upper thoracic portions of the spine. Ortopediia travmatologiia i protezirovanie. 1970;31(6):1-5.

16. Kosek E JA. Mechanisms of pain referral in patients with whiplash associated disorder. Eur J Pain. 2008.

17. Langdorf MI ZN, Khan NA, Bithell C, Rowther AA, Reed K, Anderson CL, Lotfipour S, Rodriguez R. Yield and clinical predictors of thoracic spine injury from chest computed tomography for blunt trauma. West J Emerg Med. 2014;15(4):465-70.

18. Lemming D, Graven-Nielsen T, Sorensen J, Arendt-Nielsen L, Gerdle B. Widespread pain hypersensitivity and facilitated temporal summation of deep tissue pain in whiplash associated disorder: an explorative study of women. Journal of rehabilitation medicine. 2012;44(8):648-57.

19. Moog M, Quinter J, Hall T, Zusman M. The late whiplash syndrome: a psychophysical study. Eurpoean Journal of Pain. 2002;6:283-94.

20. Nirula R PF. Identification of vehicle components associated with severe thoracic injury in motor vehicle crashes: a CIREN and NASS analysis. Accident; analysis and prevention. 2008;40(1):137-41.

21. Raak R WM. Thermal thresholds and catastrophizing in individuals with chronic pain after whiplash injury. Biological Research for Nursing. 2006;8(2):138-46.

22. Rao RD, Berry CA, Yoganandan N, Agarwal A. Occupant and crash characteristics in thoracic and lumbar spine injuries resulting from motor vehicle collisions. Spine Journal. 2014;14(10):2355-65.

23. Rotstein OD RF, Molina E, Simmons RL. Mediastinitis after whiplash injury. Canadian journal of surgery Journal canadien de chirurgie. 1986;29(1):54-6.

24. Sjostrom H, Allum JH, Carpenter MG, Adkin AL, Honegger F, Ettlin T. Trunk sway measures of postural stability during clinical balance tests in patients with chronic whiplash injury symptoms. Spine. 2003;28(15):1725-34.

25. Wallin M, I R. Quality of life in subgroups of individuals with whiplash. Eur J Pain. 2008;12:842-9.

26. Waxman SG RM. The whiplash (hyperextension-flexion) syndrome: a disorder of dorsal root ganglion neurons? J Neurotrauma. 1996;13(12):735-9.
